# Editorial and Miscellaneous

**Published:** 1862-07

**Authors:** 


### EDITORIAL AND MISCELLANEOUS.

*Researches and Observations on Pelvic Hæmatocele. By J. Byrne, M. D., M. R. C. S. E., Resident Fellow of the New York Academy of Medicine, &c. Monograph. From the Author.* 1862.

For this well written pamphlet which contains the most complete and perspicuous *resume* of the subject we recollect to have seen, combined with original observations of an important character, we return sincere thanks to the author. It fills a hiatus in American medical literature which in the author's words, for the honor of our progressive science, should no longer exist. About thirty years since, Prof. Recamier, first among the moderns at least, discovered and described this somewhat rare form of pathological lesion. Velpeau, Tilt, West, Bedford, but especially Nelaton, have since contributed from their experience, and occasional articles on the subject have appeared from various pens.

The author prefers the nomenclature Pelvic h. to " recto-uterine," " retro-uterine," or " peri-uterine," " as the extravasated fluid does not invariably select either of the locations thus indicated," a case illustrative of which proposition is given.

It occurs most frequently between 25 and 35 years, when the sexual system is in its greatest vigor, and consequently the pelvic organs more prone to congestion. Does not depend merely upon disordered menstruation, but upon some pre-existing lesion. He thinks that 80 per cent. of the recorded cases have depended on ovaritis resulting in a varicose condition of the blood vessels, softening of the tissues, &c., ultimately terminating in rupture and extravasation.

We are particularly pleased here by a suggestion in a foot note: "Varicosity of the veins may undoubtedly be a cause, as well as a consequence, of ovarian inflammation—perhaps the former more frequently. If so, would not the term *Ovarian Phlebitis* more correctly express the actual state of the parts affected?"

The pre-disposing and exciting causes are:

"1st. Inflammation of the uterine appendages and its consequences, oftentimes the primary, and by far the most frequent among the *predisposing causes* of pelvic hæmatocele. (2d.) Habitual constipation of the bowels, and morbid growths interfering with the free return of venous blood, and thereby producing a varicose condition of the vessels. (3d.) A hæmorrhagic diathesis from a disordered state of the blood. (4th.) Tubular, uterine, or vaginal occlusion, obstructing the normal secretion or giving rise to regurgitation through the Fallopian tubes. The immediate or *exciting causes* may be, (1st.) Sudden suppression of the menstrual, or a hæmorrhoidal discharge; (2d), tenesmus or violent muscular exertion; (3d), injuries by a fall or otherwise, and (4th), excessive coitus, and mental emotions tending to active congestion of the internal organs of generation.

Still another cause remains to be mentioned which might, with propriety, be classed both as predisposing and exciting, namely, *extra-uterine pregnancy*."

Then a general summary of the prominent symptoms:

"When the hæmorrhage takes place from the under surface of the ovary, or within the folds of the broad ligament, the patient, having previously suffered more than usual from pain in one or other iliac region, will complain suddenly of severe cramp in the lower portion of the bowels, accompanied or soon followed by tenesmus, and weight referred to the loins and sacrum; there may be painful and difficult micturition, and if the quantity of blood poured out be great, faintness, and even complete syncope may now take place; the skin assumes a pale or sometimes anæmic hue; the extremities become cold, the countenance anxious, pulse small and frequent, and the abdomen tympanitic, and very sensitive to pressure, particularly over the seat of the rupture. At this stage of the case, a vaginal examination will rarely fail to detect a tolerably firm and irregular tumor, somewhat painful to the touch, and situated directly behind, or to either side of the uterus."

The Diagnosis is stated to be, in many instances, extremely difficult, such acute observers as Malgaigne and Nelaton, each having reported cases of their own error. Nevertheless, in general, it can readily be made out.

The principal diseases with which it is liable to be confounded are pelvic abscess, retroversion of the uterus, dislocated ovarian cysts or fibrous tumors, extra-uterine fœtation, and possibly purulent collections. The methods are well described for avoiding mistake.

The treatment should be three-fold—*preventive*, *palliative*, and *curative*—preventing or removing the causative lesions, palliating by general and local measures according to the indications, and lastly removing the local affection. The author is evidently in favor of early puncture of the cyst by the trochar, and free evacuation of its contents, although he admits they may occasionally be absorbed spontaneously, or under the use of the ordinary methods—counter irritation, the internal use of iodine, murcury, &c., rest in the horizontal posture, and strict attention to the state of the bowels. He suggests puncture through the rectum as the most eligible, for what appear sound reasons, at least in the case given:

"(1st,) The posterior wall was thinner and more dependent; (2d), there seemed to be less danger of wounding branches of the hæmorrhoidal or other arteries—no pulsations having been noticed on this surface of the tumor; (3d), the discharge, if long continued, would be less disagreeable to the patient; and, (4th), when spontaneous evacuation of the cyst takes place, it occurs more frequently, and with no less favorable results in this locality than in the vagina."

The propriety of leaving the canula in the opening must be decided according to the place selected for puncture, its degree of tenuity or of thickening and infiltration—the cardinal principle being to secure free evacuation, with as little discomfort as possible to the patient. Examination of the recorded cases fails to convince the author either of the safety or utility of the injections usually recommended. The cases where it may become advisable are exceedingly rare.

Repeated punctures may be necessary, and he italicizes the proposition: "*I am fully satisfied that, in a retro-uterine hæmatocele of any considerable magnitude, it would be safer to penetrate the tumor twenty times than to 'always rely on absorption.'*"

The pamphlet, which our space will not permit us further to follow, concludes with remarks upon the frequency, causes, seat, and dangers of the disease. Four-fifths of the cases occur in married women. It is relatively, according to Prof. Simpson, more dangerous than pelvic cellulitis, but the author justly observes that these widely diverse affections ought scarcely to be associated ætiologically, as the remark might suggest. "*Encysted bloody tumors within the peritoneal cul de sac are extremely rare, those occupying the structures external to that membrane constitute the great majority*—say 75 or 80 per cent. of the whole." The diagnostic signs of the particular location unfortunately are unreliable, for "although a lateral position of the tumor will always indicate its subperitoneal character, yet the fact of its being central and occupying the whole posterior part of the vagina, does not, by any means, prove the contrary; and, secondly, that the position, size, or shape of the swelling—though *if intra peritoneal always central* both above the brim as well as in the vagina—possesses but little, if any, value in differential diagnosis."

We must refer our readers to the monograph itself for further details, commending it to their careful perusal.

---

*Advice to a Mother on the Management of her Offspring.* By Pye Henry Chavasse, F. R. C. S. E., &c., &c. "Lo, children and the fruit of the womb are an heritage and gift that cometh of the Lord." Reprinted from the sixth London edition. New York. Bailliere Bros., 440 Broadway.

This is a little volume of 175 pages, the remarkable popularity of which in England has led to its republication in this country. We suggest to the enterprising publishers that the value, interest, and permanent importance of this volume warrant a more durable enclosure than a paper cover. It should be in the hands of every mother, and be carefully read, re-read, and conned.

The three sections into which the treatise is divided, discuss, in the catechetical form, the management of Infancy, Childhood, and Youth. Having carefully examined the author's directions, we find them eminently judicious and sensible. The physician will be the means of great good to the families under his charge by seeing that they are put in possession of this manual. There are here and there points which we are disposed to disagree with, but taken together we find little to qualify the general commendation above given.

---

*Medical Testimony in regard to the proper Medical Treatment of Joint Diseases.* Henry G. Davis, M. D., 19 Park Avenue, New York city. Pamphlet. From the Author.

The object of Dr. Davis in the publication of this pamphlet is two-fold. First, to lay the mechanical part of the treatment before his brother practitioners, and, second, to establish his claim to the honor of its origination. "On the last point," he remarks, "that while in this country a medical fellow-townsman quietly misappropriated, without acknowledgement, the apparatus that I had freely made him acquainted with—in England there are two men fighting, even now, over the same apparatus, each claiming its invention prior to its introduction by the other, whereas, in fact, both have borrowed it from me." It appears subsequently in the paper that Dr. Lewis A. Sayre is the "fellow-townsman" alluded to. Dr. Davis adduces the testimony of Prof. Alfred G. Post, Dr. Gurdon Buck, Dr. John Watson, Prof. Raphael, Prof. Henry H. Smith, Dr. O. C. Gibbs, Dr. Parker, and others, as fully establishing his priority over all others, and especially claims to have shown and explained the apparatus to Dr. Sayre personally. His point seems pretty clearly made out.

Aside from the priority question, the pamphlet is interesting as containing diagrams of the apparatus, and plates illustrative of its successful use.

*Hand-Book of Surgical Operations.* By Stephen Smith, M. D., Surgeon to Bellevue Hospital, New York. Bailliere Bros., 440 Broadway.

This is a small octavo of 279 pages, with a flexible cover, adapted for the pocket companion of the military surgeon. From the observation we have been enabled to give it, we do not hesitate to say that it is the best adapted to its object of any work we have yet seen. It is truly wonderful, the amount of valuable information the author has contrived to get into this compact space.

The work is divided into six chapters, the first treating of minor surgery, including the titles, Instruments, Union of Wounds, Dressings, Hæmorrhage, Bloodletting, Counterirtants, Vaccination, Anaesthetics. The second chapter treats of the Wounds and Ligatures of the arteries: the third, Wounds of the Veins, and Varicose Veins: the fourth, amputations: the fifth, of Resections: the sixth, of Gun Shot Wounds, &c.; closing with a copious index.

The feature of the work is its profuse illustration, which saves at a glance pages of description. Single instruments, pocket cases, field cases, brigade cases, bandages, ligatures, incisions, and special operations—about everything is thrown into practical form which the eye can take in at once.

So far as we can judge from hasty perusal, the Author has been very fortunate in the text, elucidating the details of each part with clearness and precision.

We cordially recommend the work to our military compeers, and indeed to practitioners generally.

The war is producing something of the effect upon medical and surgical literature that the magnetic telegraph is upon general writing—great and steady condensation, with consequent increase of pith and force. Knowledge is being divested of its cumbrous and disfiguring wrappings and disguises. As Bacon wished, it is being digested to aphorisims. It will not do in these days, when a man has conceived a dozen or so of ideas, to straightway fall to making a big book about them; the greater part borrowed from those gone before, a dozen pages his own, and the rest rhetoric—all that has gone by. There *is* a beautiful statue in the great boulder of medical science, and skilful mallets and chisels are fast clipping their way down upon it.

---

*A Manual of Medical Diagnosis:* Being an analysis of the Signs and Symptoms of Disease. By A. W. Barclay, M. D., &c., &c. Second American from the second revised London edition. Pp. 451. Philadelphia: Blanchard & Lea, 1862. From the Publishers.

In the preface to this volume, the author states: "The very early period at which a call has been made for a second edition of this manual, has prevented my attempting anything in its revision beyond verbal alterations and minor additions; in its plan and details the work remains the same."

Most of the readers of this Journal probably already have "Barclay's Diagnosis" in their libraries—to them we need say nothing. But if there be one so unfortunate as not to have it, we most earnestly advise him to possess himself of the work at the earliest moment.

We know of no book which can supply its place, either to the old or young practitioner. Many a knotty case will be cleared up on reference to its pages. For perspicuity and precision, for conciseness and condensation, it is a model among medical books.

In these days of "general principles"—under which term too many shelter themselves whilst failing to ascertain precisely what is the matter of the patient under treatment—it is refreshing to read a treatise which makes want of exactness in diagnosis the next thing to criminal. For "where knowledge is a duty, ignorance is a crime." To ascertain what is really the matter of the patient, is the first great "general principle," to which all others are subordinate, a fact which we regret to say we see every day lost sight of. It is the very text of the sermon, which once being thoroughly understood, the "secondlys, thirdlys, fourthlys, application and improvement," follow almost as a matter of course. Remedies abound, and "the cry is still they come," an infinite host like the sands of the sea shore or the stars of heaven in number. And the tendency all the time is to begin at the wrong end of the matter. We are hunting for antidotes and specifics and what-not, with about as much wisdom as Crusoe would have manifested in coining moidores instead of sowing grain and breeding goats. We are filling a bottomless pitcher with new remedies, as the Homœopathists are filling infinity with infinitesimals. It is time to begin again, and if we are to take our pitcher to the fountain of Hygeia, let us see to it that we get hold thereof by the right handle. Diagnosis—diagnosis, *iterum, iterumque*, that is the thing. If we would have successful treatment any thing more than a lucky accident, this first standpoint must be gained. When Light first was, then the world and man and all animals and plants and trees were sure to follow. Without the *Fiat Lux!* these others were problematical.

For this reason we hailed the first edition of "Barclay's Diagnosis" with feelings much more akin to rejoicing than we did ———'s great volumes on Practice. Because Dr. Barclay seemed to give us the *pou sto*, with a chance to use the lever. The big volumes—chaotic, "dry rubbish shot here," with much confusion of "what is the matter"—pelted poor patients in the darkness with all sorts of missiles, many of them, even in the light, of most questionable shape.

Briefly, Dr. Barclay's work is altogether invaluable—at all events worth whole shelves of speculative treatises, and sundry dozen volumes of descriptions of wondrous drugs thrown in "to boot."

We would not be understood as undervaluing medicines or disparaging their use. Not in the slightest degree. The army should be thoroughly disciplined in the use of their weapons, and should be provided with all reasonable ammunition, but it is scarcely worth while to charge bayonet at the winds, or burn powder at the ocean waves. The whereabouts of the enemy, his strength and arrangement of his forces must first be ascertained, and then don't, after the expectant quasi homœopathic style, fire pop guns at the elephants and squibs at his iron mailed men of war, or on the other hand, in "heroic" fashion, touch off hundred pounders at fleas, and explode magazines under bubbles.

Whatever may be the facts at present with reference to Therapeutics, there is no good reason why Diagnosis may not approximate an exact science, and of this patent truth the present volume is an unimpeachable witness. The greater portion of it is, beyond debate, upon the level of true science. Many particulars still remain to be cleared up—as in other sciences. But as a clear headed, sagacious, intelligent, and faithful guide, we commend Dr. Barclay in the strongest terms.

---

*Etiology of Phthisis.*—Dr. Henry J. Bowditch, President of the Massachusetts Medical Society, in his recent Anniversary Address, enunciates, as the result of prolonged and arduous investigation, the somewhat startling proposition that the main influence begetting Phthisis, is *prolonged exposure to a damp atmosphere by continued residence in damp localities.*

To sustain this view, he adduces an "immense mass of statistical facts which he has collected from every town in the Commonwealth, and which were laid before his audience in a way to produce a most profound impression." "Some of the evidence offered," says the *Boston Medical Journal*, "was of the most surprising character; stamping upon certain portions of towns the deadly seal of the destroyer, and even attaching to particular houses an almost certain doom to the residents; and in every instance in immediate connection with the influences which, in accordance with this theory, are the principal cause." The editor confesses that in listening to Dr. B's eloquent exposition of his doctrine, he could hardly resist the conviction that his arguments were unanswerable. We coincide with his expressed determination to wait until the address is in print and its doctrines be coolly and dispassionately examined. It must be admitted that there are serious difficulties, in the shape of matters of fact and common experience, in the way of speedy reception of this great generalization. The anniversary oration before the Massachusetts Medical Society is beginning to get sensational. We shall get to looking for that yearly festival and its utterances "with feelings of no ordinary emotion."

---

*English, American, and French Hospitals.*—The effort is being made in Paris to escape the unfavorable comments of the world with regard to the larger proportionate mortality of the French hospitals in comparison with American and English, by stoutly and plumply denying credit to the statistics of the latter. It is notorious that the English have done the same by the Americans for a long time.

M. Briquet, the French champion, says that he believes American statistics less than all the others. He illustrates the folly of trusting such evidence, by alluding to M. Malgaigne's remarks with reference to the Massachusetts Hospitals—"*Now,*" says M. Briquet, "*there is not a town in America which bears that name!*" What *will* the "Hub" do about it?

---

*Havelocks.*—P. A. O'Connell, Surgeon 28th Massachusetts Volunteers, in a communication to the Boston *Medical Journal*, thus speaks of the noted "Havelocks:" "On the Potomac, last summer, they were found of some use in cleaning guns, indeed, but in most cases it is respect alone for the giver that prevents one from turning them to use in some such way—for they add to the weight of the cap, without adding to one's comfort. If they could be made so as to wear without the cap, they might become of some service."

It would seem that the poor Havelocks have had their day.

---

*The Quinine Prophylaxis.*—Drs. Gray, Homans, and Hodges, the surgeons specially detailed by the Governor of

Massachusetts to visit the army in Virginia, report to the State Surgeon General, and the following sensible remarks we extract:

"The diseases were principally of a typhoid character, genuine typhoid fever, typhoid pnuemonia, dysentery, diarrhœa, acute and chronic rheumatism. The type of all of them may be said to have been generally mild, in spite of the great fatigue, and, to many, exhaustion, from work in the trenches by night and corduroying the roads by day, with other labor incident to military encampments, and frequent exposures to long-continued, cold, north-east rain storms.

"*Malaria was said to be acting powerfully, and therefore quinine must be administered in large doses. The ill effects from this large dosing was found to be much greater than that from any supposed malarial influence. The improvement in every instance where the quinine was either entirely stopped or given in greatly reduced quantities, was too marked and too continued to leave a shadow of a doubt as to the exciting cause of the persistent head ache and diarrhœa. The good effects of stimulants, brandy or whiskey, was immediately seen, when we had some to give.*"

Is not this monstrous "quinine prophylaxis" nonsense about "played out?" The word of its promise is not even kept to the ear, and the humbug proves dangerous as well as expensive. We are thus every day becoming more thoroughly convinced (if it were needed) of the truth of the propositions we laid down upon this subject early in the war. Visionary theories will not abide the touch of experience and truth.

---

*Williamsburg.*—It is estimated that there were some thirty or forty thigh amputations after the Williamsburg battle. Primary amputations have proved more successful than secondary.

---

*The Military Department.*—Some little matters about the medical department of the army seem to demand a change. Thus, Dr. A. H. Baker, editor of the Cincinnati *Medical and Surgical News*, writes to his Journal from Pittsburg Landing, April 13th:

"We arrived here about 10 A. M. to-day, and in less than thirty minutes, witnessed an amputation at the *shoulder joint;*

the operation was performed by a surgeon on one of the boats from Cincinnati. The poor fellow will only enter the portals of eternity a little earlier than if he had been permitted to fight his last battle undisturbed. I make this remark because I have been over the field of battle, and have seen enough to convince me that our success with the knife, at this period, must be a failure, and for one, I am determined to depend upon *conservative surgery* as affording the best chance for *life*, and, surely, for *limb*. I am fully satisfied that many of the brave men who have been wounded in battle since the commencement of this unholy war, have been sacrificed upon the altar of a mistaken ambition for fame, as an operator. It does seem that the soldier is a legitimate subject for horrible abuse, that the moment he is entitled to the name, he becomes a soulless mass of animal matter, only fit to be shot at, and when he escapes death from the immediate effects of the minnie and starvation, he can find it under the knife of the *surgeon;* if such we can call the man who is willing to lay down the advantages of a scientific education and take up the character of a worthless tyro, or a paid demonstrator. But, Doctor, I do not wish you to misunderstand me, and to suppose that I have reference to *army surgeons*, for, my dear sir, they have been outrageously abused by the press, and that, too, without a knowledge of the situation in which these men are often placed. Let me illustrate by the facts which present themselves here. On Sunday the 6th, the Confederates took possession of all our stores, and after the battle was ended, our surgeons found themselves without the necessary means for effective treatment, and thus the wounded were, in many instances, neglected until a *new set*, with supplies and ready hands, made their appearance to try their skill and to witness the result of *secondary* amputations, to which my remarks, at present, refer.

One thing more presents to my mind a horribly mistaken philanthropy; it is the *removal* of the wounded from the field of devastation. The whole thing is wrong, and comes in as the *finishing stroke.* Behold a man severely wounded, a bone broken, much exhausted, and just in a condition to convalesce, with care; he is just taken up by *carriers*, and on his way to the boat. Hear him complain at every jar, and see him nearly fainting when laid upon deck; here he must undergo various processes, such as washing, dressing, etc., etc., all done in a hurry, no time to wait; then he is *fixed* upon the boat for a trip, and is kept alive by stimulation until ready to start, when he is again subjected to great suffering, as his constant moans will tell, by the shaking of the boat. But if he lives, his sufferings do not end here. When he arrives at our landing, then he will be again brought under the carrying operation, or some other, perhaps more dreadful, when, at last, he will be deposited in one of our military hospitals, there to end an existence rendered miserable by the mistaken kindness of a philanthropic people.

"The question may be asked, what shall be done? I am free to answer, build temporary hospitals on the ground, or near the battle field, and there let them be supplied with everything necessary to afford the best possible chance for recovery. By this plan, the cost would be less, and the attention better, for the reason that changes would be unnecessary; as it is, those under the care of physicians on the boat, must pass into other hands upon their arrival at the point of destination, which may result in change of treatment to the disadvantage of the patient."

The medication of the wounded and otherwise invalided soldiers, also requires reform. From the highest authority, we have it, that on one of the hospital transports this was the state of facts. The invalids having been received, they were put under the care of the medical gentlemen on board, and a well known physician and apothecary of this city was detailed to dispense the medicines. Curiosity led our informant to look over the first prescriptions sent up to the dispenser. It is a positive fact that of the first twenty-eight, *ten* contained Hydrarg. Subchlorid and Pulv. Ipecac comp.; ten contained Hydrarg. Subchlorid and Pulv. Ipecac, and eight contained Tinct. Verat. Virid. How long this continued he is unable to state, for at that point he ceased his investigation. To his credit, be it said, that he suggested to the apothecary the benevolent substitution of Carb. Sodae for the Hyd. Subchlor. and Brandy for the Verat. Virid.

We have hesitated before giving this humiliating record—for humiliating it is in the last degree. There are some men who cannot distinguish an attack upon abuses from an attempt to overthrow. Some months ago, when we remonstrated in mild terms against the frequent salivations at Cairo, weakkneed and tender-footed gentlemen feared it might "hurt the profession" to have the facts known. When almost an entire transport load of salivated soldiers is received at once on the Tennessee, and such facts as we have given above are published, we suppose there will be another flutter among the birds. But it cannot be helped—"what we have written is written," and we have only put it down to denounce the enormity of the treatment. It is simply an outrage upon scientific medicine. It is just such things as this which have built up the fortunes of the quacks of every color and cognomen. We know a man in this city, calling himself a "regular physician," who used to teach parents to feed their children calomel on their bread and butter! The result has been that the *surviving* members of the families who once employed him, are homœopathists. About the last of his patients, to our own knowledge, was murdered by the same *infernal* treatment, not two years ago. And those who cultivate medicine as a science, and endeavor to practice according to its precepts as such, have to share the odium which these excrescencies, or worse, upon the medical body bring upon it. Medical societies and associations may pile their constitutions and codes heaven high, and yet not escape the effect.

We have, on good professional authority, been informed that in one camp, which has numbered its thousands, the general direction given by the ranking surgeon was that, whenever a patient's tongue tended to become dry and red, he should at once be given *Donovan's Solution*, in large doses. This was the sole test and criterion of judgment. The mortality bills at that post were high, but it is believed that we shall have some statistics with reference to the power of that peculiar specific, by and by—showing that of the many who took it, some recovered, and thus a new leaf in medical arithmetic.

It seems to the writer about time that this sort of thing should come to an end; and the way seems clear to the accomplishment of that desirable result.

The army and navy must have educated medical attendants, for the lives of soldiers and sailors are of immediate necessity for the preservation of the honor and existence of the nation. And the medical man must be educated and instructed up to the level of the times—for Medicine has progressed at an equal step with other sciences, and the man who does not keep step with it, must fall into the rear. It is no discredit to the great medical army to say that its infirm members and valetudinarians are behind. These invalids must " post up," or take the consequences. The time has come when a man can no more cover up his ignorance under his profession than, as we have elsewhere observed, under his nationality—he must stand or fall according to his knowledge, and power of applying that knowledge to the business in hand. All surreptitious props must sooner or later fail. The war is pulling down several.

---

*Acute Tropical Dysentery.*—In the *Edinburgh Medical Journal*, Dr. R. W. Cunningham, Assistant Surgeon to H. M. 4th Bengal Europeans, records his experience in the use of Ipecacuanha in larger doses than those ordinarily employed in the treatment of dysentery. His plan is on admission of the patient, to apply a large sinapism over the epigastric region, and then exhibit thirty minims of Tinct. Opi., to render the stomach tolerant of the remedy. Half an hour or an hour afterwards, from a drachm to a drachm and a half of powdered Ipecacuanhais given at once. It produces considerable nausea, but rarely vomiting, although the latter may occur under one or two hours afterward. General sedation results, and a copious perspiration breaks forth. The countenance loses the expression of suffering; the tenesmus and abdominal pains subside, the stools are lessened in frequency or cease for twelve or twenty-four hours; the pulse become full and soft. A considerable degree of languor and depression naturally follow, but the next evacuations are unaccompanied with tenesmus, free from blood and mucus, and hold in suspension small masses of fœces. A repetition of the dose may sometimes be necessary. Chicken broth and arrow root are the only diet allowed. If any tendency to debility is subsequently manifested, a few doses of Infusion of Chiretta with Nitro-Muriatic acid are sufficient to complete the cure.

The *rationale* of the process is sufficiently clear, and the writer's exposition does not require repetition. He states that relapses are less liable to occur than after the other methods of treatment adopted in the army. He suggests this method as peculiarly valuable at the outset of the disease, and as army surgeons have this advantage over practitioners in civil life, that they can take the disease under treatment in its first stage, it is especially valuable to them.

We may remark that several of our correspondents in the army, who have tried this *old* practice, speak very highly of it, but say that neither Ipecacuanha nor any other medicine they have found will prevent the return of abdominal disturbance in those who persist in the constant violation of the most simple and well known rules of health. One surgeon, almost with tears in his eyes, confessed that the leathery meat fried in tallow, and half cooked beans, would make his men sick notwithstanding quinine prophylaxis, and the swallowing of fragments of hard bread, as ostriches do nails, to favor digestion.

---

*About "Nationality."*—A correspondent from the seat of war, a month or two since, took occasion to reprehend the inefficiency or ignorance, or both, of one or two medical men, at the time in charge of a hospital transport on the Tennessee river. It happened that the aggrieved individuals were foreigners of a particular "nationality." We believe our correspondent spoke of them as "Dutchmen"—precisely as he would have spoken of them as Frenchmen or Yankees, if they had happened to be scions of either of those nations. By a dodge, adroit enough for a slippery politician or wily lawyer, the army doctor has slid under the cover of his "nationality," and discharges upon us and our correspondent a hat full of wrathful *Deitsch* expletives through a German paper in this city, among which our limited acquaintance with the language enables us to detect "KNOW NOTHING" in startling prominence.

We beg leave to assure our indignant friend that the cuttle fish plan will not work. Like the ostrich, he may hide his head (under his nationality if he chooses,) but he cannot hide that other prominent portion of his person, which on the occasion alluded to by our correspondent, was so liberally displayed.

No man surpasses our correspondent or the editor of this Journal, in respect and admiration for the German nation, its magnificent attainments, its profound scholarship, its gigantic intellectual labors. The world could not spare Germany, and the great thoughts, which move the race so wonderfully at the present day, owe their development more perhaps to the German mind than any other. But we shall enter into no needless panegyric. Among our subscribers, and among our personal friends, we are proud to reckon scores of that nationality, who in every thing which characterizes the high minded professional gentleman and accomplished scholar, "are first among their equals," of any nationality.

We simply toss back the epithets so lavishly scattered by this army surgeon, (Heaven save the mark!) as totally inapplicable in our case—the coat fits in no particular. Our educated German friends have not the slightest desire to have ignorance and pretension covered by their nationality.

It seems this army doctor's horse stumbled and sprained the rider's ankle, which, although it did not prevent the doctor's walking with scarcely a limp, does enable him to assert that he is suffering from wounds received in the defense of his adopted country, and to a certain extent prevents his emptying the remaining full vials of his wrath upon our correspondent's devoted head. We await with trepidation his recovery from his *wounds*. Meanwhile let him ameliorate the tedium of his hours whilst awaiting convalescence, by ruminating upon this—our correspondent assures us that he is prepared to prove his former statements, and, if necessary—*more too.*

We candidly advise our wrathful friend to be content with the laurels he has already won, and allow our friend, the ex-Governor, the use of his valuable columns for more profitable news or discussions.

---

*Carbonate of Ammonia in Scarlatina.*—Dr. Spooner, in his very sensible essays, to which we alluded last month, observes with reference to the great claims made for Carb. Am. in the treatment of Scarlatina, (referring particularly to Dr. Peart's work,) that there is no doubt a valuable truth contained in the accounts put forth, but, unfortunately, the statements are too extravagant, too unqualified, and allowing no exceptions. We extract the following, as containing sound thought as well as practical directions with reference to this particular agent:

"During many years of extensive practice among children, we are assured there was no death; in hundreds of consecutive cases, in every *form* and *stage* of the disease, the ammonia was given with uniform success. Thus the account promises too much, more than can be realized. It promises more than can be realized from any known remedy. We have no remedy of which it can be said, it will never fail. Vaccination does not always prevent small pox; opium does not always relieve pain; mercury does not always cure syphilis; nor does cinchona always cleanse the system of the miasma of intermittents. All that we can say of any medicine is, that it is useful, and the best we have for a specified purpose. If, encouraged by the early accounts of this medicine, a physician should be induced to use it, there is more than an equal chance that he will be disappointed; not, however, from want of power in the medicine, but, as I shall presently show, from a want of knowledge of the proper way of using it. Thus, as it too often has been, these physicians, in their earnestness to recommend a valuable medicine, did much to prevent a fair trial of it by others.

Another reason is to be found in the qualities of the medicine itself. Ammonia is a difficult medicine to give to children; and often requires much decision on the part of the physician and energy on the part of the nurse to secure for it a fair trial; and when there is ulceration in the throat, it is impossible to give it. This difficulty is a serious obstacle to the use of this remedy, especially in the hands of physicians who are skeptical in regard to the benefit of any medicine in scarlatina.

A third reason is to be found in the different ways of using the ammonia by different physicians. This is the most important reason. Indeed, this is the chief cause of the difference of opinion among physicians in regard to the value of many medicines. Different physicians use the same medicine in different doses; or they give it in *different stages* of the same disease, and very naturally come to different results. The right stage for giving a medicine is a matter of quite as much importance as the size of the dose to be given. It may require many years after a medicine has been brought into notice, before the precise limits of its usefulness can be defined —or the best way as to form and quantity of administering it, or the right stage for giving it, shall be determined. In the mean time, there may be every variety of opinion in the profession as to its usefulness. A striking example of this can be found in the history of cinchona as a remedy.

The bark of cinchona was first brought into notice as a remedy for fever and ague by a physician in 1632. For a series of years it encountered the prejudice of the profession, and, it is said, it would have fallen into oblivion had it not been for the activity of the Spanish Jesuits. Given to the Arch Duke Leopold, of Austria, at the commencement of the cold stage of an intermittent fever, it produced little or no effect, and consequently it was declared to be useless. Given to an alderman of London, and he died while using it, and it was condemned as a dangerous medicine. Oliver Cromwell was sick with fever and ague, and languished and suffered and died from it; and his physician was afraid to use the bark in consequence of its dangerous effects in similar cases. At length Sydenham, who was at first opposed to its use, candidly watched its effects, became convinced of its power, determined the quantity and frequency of its dose, and that the time of giving it was between the paroxysms, and not during them, as had been the practice.* Thus it appears that a valuable remedy may fail from error in using it. This remark is applicable to the carbonate of ammonia in scarlatina. Physicians have given it without knowing how it should be given, and consequently have often failed to obtain good results from it.

In using the subcarbonate of ammonia, there are three rules to be observed:

1. Give it as early in the disease as possible.

* Good's Study of Medicine, Vol. II, p. 130.

2. Give it in as large doses as the patient can bear, and repeat it every two, three or four hours, according to urgency of symptoms.

3. Continue it until there is improvement of the symptoms, which can usually be seen on the second or thir day.

The first rule is of paramount importance. I am confident that some physicians have failed in the use of ammonia, from not observing this rule. With our present knowledge, we cannot define the limits, as to time, of the usefulness of this medicine. So far as I have observed, its effects have been most striking when given at the very onset of the disease; and in a few cases, when forty-eight hours had elapsed before commencing the use of the medicine, I have seen no benefit from it.

The usual dose of the medicine is from three to five grains, given in simple syrup, or in water saturated with sugar. There is a serious difficulty in regard to the dose of this medicine, owing to a great difference in the article itself, as received at different times from the apothecary. When first made, the subcarbonate of ammonia, or, as the chemists call it, the sesqui-carbonate of ammonia, is solid and semi-transparent, giving a strong smell and taste of ammonia. On exposure to the air, it effloresces, and is converted into a bicarbonate, losing its ammoniacal smell and taste. In its first state it is too pungent; and if kept too long, it *may lose* its specific power. The article that I have used, has had the smell and taste of ammonia, but not so strong as to render it difficult to swallow.

As to the third rule, I have seen decided improvement in little more than twenty-four hours, when less than 30 grains had been given; and I have continued its use until more than two drachms have been taken. Improvement in most cases will take place on the second or third day, when the medicine has been given early on the first day of the attack. In one case, in which the medicine was not commenced until the second day, it was continued through five days, and with decided benefit. At one time I thought that the smell of the ammonia in the urine might be taken as an evidence that it had produced its specific effects upon the system. In several cases this odor has been well marked, and has compared in time to the improvement in the symptoms. But subsequent observations have persuaded me that improvement often takes place without the ammoniacal smell.

It has not been my practice to give the ammonia in cases which at the beginning were mild; although I have sometimes had reason to regret this course, in consequence of these patients having suffered from troublesome sequelæ, which the ammonia would have prevented.

The patients to whom I have given this medicine, have, for the most part, presented the following symptoms: Convulsions, or distressing vomiting, with anxiety, prostration of strength, and quick pulse. Convulsions almost uniformly usher in cases of a violent character; the other symptoms mentioned are usually precursors of severe cases.

---

*Deodorizing the Breath and Cleansing the Teeth.*—We find the following formulæ, which may prove occasionally convenient, in the *Druggist's Circular.* It is important that the Hypochlorite of Lime employed should be fresh and good, coming up to the standard of the pharmacophœia:

Dr. Angelot's Preparations.—The following preparation has been employed by Dr. Angelot, of Briancon, in the treatment of ulceration of the gums, a very frequent complaint with soldiers: Take of hypochlorite of lime, from 10 to 25 grains: mucilage of gum-arabic, 1½ to 4 drachms; syrup of orange-peel, 1½ to 2 drachms. Mix thoroughly. This mixture is employed as a lotion to the ulcerated gums.

Pastilles of Hypochlorite of Lime.—Several formulas for the preparation of the pastilles have been successively published; these preparations have the advantage over those which are liquid, of being more easily transported.

*First Formula.*—Take of hypochlorite of lime, 7 drachms; sugar, flavored with vanilla, 3 drachms; gum-arabic, 5 drachms. The pastilles are made *secundum artem*, so as to weigh from 10 to 11 grains. Two or three of these pastilles are sufficient to remove from the breath the diagreeable odor produced by tobacco smoke. The pastilles thus prepared have a gray color, and become quite hard; if pastilles of a white color are required, the following substances are employed:

*Second Formula.*—Take of dry hypochlorite of lime, 20 grains; pulverized sugar, 1 ounce; gum tragacanth, 16 grains. The hypochlorite of lime is triturated in a glass mortar, and a small quantity of water is poured upon it; it is then left to repose, decanted, and a second quantity of water added; the two liquids are filtered, and the gum and sugar added so as to form a paste. This is divided into pastilles, weighing from

12 to 16 grains. If it is desired to aromatize the paste, one or two drops of any essential oil may be added; the oil should be added to the sugar and gum before the paste is formed.

Deschamps' Pastilles.—Take of dry hypochlorite of lime, 2 drachms; sugar, 8½ ounces; starch, 8 drachms; gum tragacanth, 1 drachm. carmine, 2½ grains. The pastilles should be made so as to weigh about 2½ grains; five or six may be taken in the space of two hours. By employing starch in the preparation of the lozenges, Deschamps wishes to prevent the yellow color which they would otherwise assume.

Deschamps' Dentifrice for Removing the Yellow Color from Teeth.—Take of dry hypochlorite of lime, ½ drachm; red coral, 2 drachms. Triturate well and mix thoroughly. This powder is employed in the following manner: a new brush is slightly moistened, then dipped in the powder and applied to the teeth. According to the author, a few days use of this powder will produce a marked alteration in the appearance of the teeth, which will acquire a white color.

---

*Nitric Acid Fumes.*—Two men, employed in a chemical manufactory at St. Denis, were found dead in the street soon after leaving their work. Their bodies were removed to the Morgue for examination. The medical opinion was that their death was due to the inhalation of the fumes of nitric acid. It is suggestive of further observations and precautions with reference to the large class of operatives in manufactories, telegraph offices, &c., who are constantly more or less exposed to the fumes of this powerful agent. It would seem that measures might readily be adopted to prevent deleterious or dangerous results.

---

*Alveolar Abscess.*—W. H. Atkinson, M. D., in the *Dental Cosmos*, writes that molecular degeneracy *must* precede all idiopathic formations of pus. In traumatic lesions where a foreign body is forced within the tissues, a kind of artificial abscess is formed, an exudate partially or completely encysting it, whence, if soluble, the foreign substance is gradually absorbed, or reduced to its smallest compass. If insoluble, and located not too near centres of motion or certain important organs, it may remain inert—the abscess spontaneously cured without a rupture of the cyst. But when the molecular degeneracy is more extensive or pervading the cyst is less, or not at all defined, pus may burrow every where "around and beneath muscle, tendon, blood-vessels, and nerves, until the poor patient is literally one bag of illy formed pus and broken down adipose tissue, between the deep and superficial layers of fasciæ.

Cases of what is popularly called "ague in the face," are closely allied to this sort of molecular degeneracy, but recently induced by excessive fatigue, bad feeding, or deficient food, or food of poor quality, cold, or excess of indulgence in the venereal function, or depressing afflictions of the affections," and are very apt to be tedious and harrassing to patient and practitioner.

This degeneracy it is our first duty to correct by controlling the aberrant forces and restoring nutrition by better regimen and morale. General sedation of the main trunks of the vascular system, and local applications, cold, warm, or hot, to the tumefied face are recommended. These may prevent altogether, or at least localize, the formation of pus. When the abscess points, open it and dress it morning and evening for a few days with equal parts of pure creosote and tincture of iodine, gradually extending the time until the discharge resembles the white of an egg, then the treatment may be suspended, but the case should still be watched as relapses are not unfrequent. A bit of cotton, or of cork, spunk, or soft wood, on the end of a knitting needle, will be found a convenient means of dressing the cavity—leaving the pledget in the part. If the depth is miscalculated and the cotton projects, cut it off carefully, and apply over it, between the wound and the buccal walls, another pellet of cotton, wet in Tinct. Arnica, diluted with four parts of water.

When it is remembered how often these so often neglected abscesses lay the foundation of caries and even general necrosis, or other diseases of the maxillæ, it will be seen that too much care can scarcely be exercised to prevent such disastrous results.

---

*Cerebro-Spinal Meningitis.*—Occasional cases of this fearful form of disease have recently appeared in this vicinity, and also in some parts of Iowa, Missouri, Kentucky, and Tennessee. It is not too much to say that any practitioner, who has passed through an epidemic of it, had rather encounter almost any other form of disease to which the human system is liable. Unfortunately, the diagnosis and morbid anatomy are much better understood than the appropriate treatment. The very best practitioners we have ever known, have had little reason to plume themselves upon their success with it. It is safe to say that its treatment is, as yet, as unsatisfactory as that of Asiatic Cholera—the results are, on the large average, even more fatal.

One error should be avoided—it is not allied in nature to *Congestive*, *Pernicious*, or *Malignant* "Malarious fever," as ordinarily described in the books or understood in practice. The "Pernicious Remittent" of Wood, and other authors, is as distinct from it as a severe summer diarrhœa is from Asiatic Cholera. It is not a less or more severe form of remittent, or intermittent, fever, but a totally distinct affection, and should not by any means be confounded with it. There may be chill, and coma or delirium in each, but it is the loosest possible observation and judgment that can be decided by these coincidences.

We do not propose to rehearse the symptoms or special diagnostic marks, as we could add but little to what the text books contain, but merely wish to direct the attention of the young men of the profession to the well defined distinctions between the two forms of disease, recently, we regret to say, most remarkably ignored by the published *decision* of a medical gentleman who had no right to commit so grave an error in diagnosis.

The Pernicious, Congestive, or Malignant form of Intermittent or Remittent fever, although attended with the gravest symptoms and most dangerous without treatment, is most singularly amenable to the ordinary well known treatment, and *is readily controlled.*

Cerebro-Spinal Meningitis, on the contrary, presenting some symptoms in common with the foregoing, yet affords many strikingly distinct symptoms by which it may be positively diagnosed, and, as before remarked, is unfortunately one of the most intractable of diseases with which we are called upon to cope.

---

*Climate of California in Phthisis.*—Dr. James Blake, continuing his series of articles on the climate of the mountain ranges of California in relation to the treatment of Phthisis, has brought together a table of thermometrical observations which strikingly illustrate its advantages. The data go to show, however, that the climate is very diverse in different parts of the State. Thus, the climate of the Sierras very closely approximates that of the Sacramento valley, which is far less favorable as a summer residence for consumptive patients than that of the Coast Range. During the month of July, the most agreeable month for living in the open air, the range of temperature on the Coast Range was never below 57 deg., and never above 82 deg., with a variation during the twenty-four hours never exceeding 20 deg.

Dr. B. observes that the temperature is by no means the sole advantage, for together with this the atmosphere is dry and bracing, and the sky unclouded for months together. The last summer there was no rain from the beginning of May until the end of November, until which latter time his patients remained abroad. About the middle of September they began to descend towards the plain. The lower ranges offered a climate well adapted to the early summer and late autumn months. The highest elevation of the camp was about 4,500 feet.

Game is abundant, and thus the camp is well supplied. At the coming on of the winter and rainy season, he advises patients to seek a similar climate to the California summer in the highlands of Mexico, which may easily be reached by steamers and sailing vessels from San Francisco via Mazatlan three or four times a month, and from thence by a short land trip of eighty miles they can gain the regions of perpetual summer. The country, he remarks, is well settled, and as large mining operations are carried on there by our own countrymen, all sorts of supplies can be secured. Dr. Blake concludes his paper by asserting his belief that in no other part of the civilized world can such favorable conditions be found for treating Phthisis as on that coast. That it cannot be cured by drugs, is a fact patent to the profession. That it can be cured by out-door life in the mountain air, is a fact of which he has seen abundant proofs, and which others in crossing the Plains have had opportunities of verifying. His own opinion is that a large proportion of cases of Phthisis will be found to yield to one or two years open air treatment, and to carry out that plan the California Coast Range is the best known locality.

---

*Antagonistic Effects of Opium and the Mydriatics.*—Several cases have recently appeared illustrative of the correctness of Dr. Lee's position in the paper on this subject in the American Medical Journal. Poisoning by Belladonna and Stramonium has been speedily relieved by the exhibition of full doses of Morphine; and *vice versa*, poisoning by opiates has been controlled by large doses of Belladonna. An incidental practical suggestion is, that it is scarcely worth while to mix these articles in the same prescription, provided it is intended to secure a decided result.

---

*Iodide of Potassium.*—The Times & Gazette remarks that Iod. Potassii is not only maintaing its reputation as an alterative, but really gaining in general favor. Although very valuable in rheumatism and in securing absorption of inflammatory products, it is yet the most strikingly beneficial in tertiary syphilis. The old bugbears—wasting of the breasts and testicles—are now never heard of, and *iodism* is certainly exceptional. Eight and ten grains are now with many an ordinary prescription. Dr. Gull and Mr. Paget strongly commend the addition of ammonia as enhancing its activity. Some surgeons combine it especially in brain and nerve disorders, with Tinct. Nucis Vomic.—it is asserted with great advantage.

---

*Arsenic in Ophthalmia.*—Mr. Critchett commends Arsenic in certain chronic forms of Ophthalmia, especially in young persons. It is chiefly useful in intractable cases of purulent Ophthalmia, attended by intolerance of light and a feeble and irritable state of the system generally. It may in these cases sometimes be well combined with iron, or the steel wine. For a child of five years, he orders a minim of the Liq. Pot. Arsenitis in a teaspoonful of the steel wine. Upon which suggestion we shall make no comment, but subjoin a *feu de joie* from Dr. O. W. Holmes:

The more you examine the structure of the organs and the laws of life, the more you will find how resolutely each of the cell-republics which make the *E pluribus unum* of the body maintains its independence. Guard it, feed it, air it, warm it, exercise or rest it properly, and the working elements will do their best to keep well or to get well. What do we do with ailing vegetables? Dr. Warren, my honored predecessor in this chair, bought a country-place, including half an old orchard. A few years afterwards I saw the trees on his side of the fence looking in good health, while those on the other side were scraggy and miserable. How do you suppose this change was brought about? By watering them with Fowler's solution? By digging in calomel about their roots? Not at all; but by loosening the soil around them, and supplying them with the right kind of food in fitting quantities.

Which we think hits that case, and some others, exactly.

---

*Acupressure.*—Dr. E. L. Cooper, in the San Francisco

Press, writes the obituary of this surgical humbug, so popular with certain journalists a couple of years ago. We predict a similar early death for a similar humbug, to relieve local pain, which has gained much eclat within the same period.

---

*Ovariotomy.*—M. Nelaton, finding this operation condemned on what appeared to him unsubstantial grounds by the French surgeons, recently visited England on the invitation of Dr. I. Baker Brown, to observe the results of his operations. He found that Dr. B. had performed the operation with marked success. His conclusions, as given by a correspondent of the San Francisco Press, are as follows:

1. In presence of mutilocular cyst, at its debut, before it has occasioned grave accidents, I should abstain from the operation. Because there are examples of the tumor remaining stationary for years, without causing even distress.

2. I should also abstain, in case of an old cyst, which has undermined the constitution of the patient.

3. I think the opportune moment to be, when the cyst commences to swell up rapidly, and the danger of serious accident is imminent. Then, and then only, would I operate.

---

*Large Doses of Morphia.*—Mr. Parish, of Philadelphia, having some time since called attention to the fact that Morphia is frequently given in much larger doses than formerly, the proposition was called in a question by another apothecary. The Druggist's Circular very properly observes: "The simple denial on the part of our other correspondent does not remove the fact that some doctors prescribe the narcotic in such doses, any more than it acquits a thief if he brings ten witnesses who have not seen him commit the crime against one who witnessed it."

What *does* the Circular mean? Are not such "comparisons *odorous*"—"very tolerable and not to be endured?" The way our contemporary puts the case annoys us, but of the fact there can be no question.

The reason is because physicians now realize that pain is by no means salutiferous and should be put down: because it is found that many of the supposed beneficial effects of calomel and other drugs were really due to the opiates with which they were combined; because they have found, practically, that opium, or morphine, its principal exponent, is competent to produce the same effects, when properly managed, formerly expected only from bloodletting, or other evacuants, and powerful sedatives. The nonsense about its "locking up the secretions," is pretty effectually exploded, and almost everybody knows it ranks next to and sometimes above quinine as an antiperiodic. These are only a few of the reasons why.

---

*Adulteration of Milk.*—It is stated that Borax is employed to prevent milk from turning sour, and also to impart more consistence, so as to appear more creamlike.

---

☞ Subscribers will please direct business or money letters to Dr. INGALS, Box 14, Chicago, Ill.

